# Wave Frequency Effects on Damage Imaging in Adhesive Joints Using Lamb Waves and RMS

**DOI:** 10.3390/ma12111842

**Published:** 2019-06-06

**Authors:** Erwin Wojtczak, Magdalena Rucka

**Affiliations:** Department of Mechanics of Materials and Structures, Faculty of Civil and Environmental Engineering, Gdansk University of Technology, Narutowicza 11/12, 80-233 Gdansk, Poland; magdalena.rucka@pg.edu.pl or mrucka@pg.edu.pl

**Keywords:** Lamb waves, scanning laser vibrometry, adhesive joints, non-destructive testing, damage detection, excitation frequency

## Abstract

Structural adhesive joints have numerous applications in many fields of industry. The gradual deterioration of adhesive material over time causes a possibility of unexpected failure and the need for non-destructive testing of existing joints. The Lamb wave propagation method is one of the most promising techniques for the damage identification of such connections. The aim of this study was experimental and numerical research on the effects of the wave frequency on damage identification in a single-lap adhesive joint of steel plates. The ultrasonic waves were excited at one point of an analyzed specimen and then measured in a certain area of the joint. The recorded wave velocity signals were processed by the way of a root mean square (RMS) calculation, giving the actual position and geometry of defects. In addition to the visual assessment of damage maps, a statistical analysis was conducted. The influence of an excitation frequency value on the obtained visualizations was considered experimentally and numerically in the wide range for a single defect. Supplementary finite element method (FEM) calculations were performed for three additional damage variants. The results revealed some limitations of the proposed method. The main conclusion was that the effectiveness of measurements strongly depends on the chosen wave frequency value.

## 1. Introduction

Adhesive bonding is one of the effective methods for joining elements in metallic structures, besides welding, riveting, and bolting [1]. It has dozens of applications in the aerospace, machine, automotive, military, and electronics industries [2]. Structural adhesive joints have numerous advantages in comparison to other joining techniques. Firstly, adhesives do not interfere with the structure of adherends (joined elements), which is what happens in bolted joints (openings weakening joined parts) or welded joints (internal stresses after welding). Moreover, gluing enables the creation of heterogenic connections, especially when welding or hole drilling is forbidden. There are also some disadvantages; from these, among the most significant is high vulnerability to accuracy in the processes of preparation and manufacturing. Particularly, the most important issues for the strength of the joint are the accurate surface treatment [3] and the protection against any contamination [4]. Any inaccuracy may lead to the formation of kissing defects or voids [5,6]. Their presence can cause a significant decrease in the strength of the joint and, as a result, its failure. The problematic issue is that kissing defects are not detectable in the visual assessment, because of their existence in the internal structure of the joint. This creates the necessity of application of non-destructive testing (NDT). There are a number of promising methods that have also been successfully applied for damage identification in adhesive joints, using ultrasounds [7,8,9], thermography [10], radiography [11], laser-induced breakdown spectroscopy [12], or electric time-domain reflectometry [13]. These methods are the basis for structural health monitoring (SHM) systems that provide a real-time evaluation of analyzed structures of different types, such as bridges [14,15,16], tunnels [17], or marine structures [18]. Nowadays, SHM strategies are becoming more and more popular for composite materials also [19,20,21].

The guided wave propagation phenomenon is commonly used for damage identification in structures of different types (e.g., [22,23,24,25,26,27]). Lamb waves are a specific type of guided waves that propagate in plate-like elements. It is worth noticing that they are multimodal; i.e., in general, an infinite number of different modes (symmetric and antisymmetric) can propagate in each medium. Another significant feature is the dispersive nature, which means that wave characteristics such as the wavenumber and propagation velocities of each mode are frequency-dependent. These properties make the question of wave propagation a complex problem. For certain frequency ranges, some modes do not propagate, whereas for a different range, the same modes can travel with certain velocities; thus, they influence the wave propagation. For this reason, the appropriate choice of the excitation frequency is an essential issue for the effectiveness of obtained results. High sensitivity to any disruption of geometry and changes in material properties create many applications of guided waves in non-destructive diagnostics of existing structures. Previous studies prove their usefulness for the identification of damages of different types, such as cracks in metallic beams and plates [28,29] or delamination and flaws in composites [30,31,32]. With regard to the adhesive joints, guided waves were efficiently used for the identification of disbond areas in the single-lap joints of plates made from different materials. Ren and Lissenden [7] detected damaged areas in the adhesive film in a CFRP (carbon fiber reinforced polymer) plate stiffened with a stringer using the adjustable angle beam transducers. Nicassio et al. [33] analyzed debonding in an adhesive joint of aluminum plates using piezo sensors. Sunarsa et al. [34] used air-coupled ultrasonic transducers to detect debonding and weakened bonding areas of different shapes in adhesively bonded aluminum plates. The time of flight of the measured signals was estimated with the support of the wavelet transform. Parodi et al. [35] analyzed numerically and experimentally wave propagation in a wall of a composite pressure vessel with flaws in the interface between the aluminum layer and CFRP coating, considering the excitation frequency in a range of 20 to 100 kHz. Ultrasonic waves are also successfully used for the evaluation of adhesion levels between adhesive and adherends. Gauthier et al. [36] analyzed the influence of different adherend surface treatment methods on the guided wave propagation in a single aluminum plate covered with epoxy-based adhesive. Castaings [37] considered a contamination of the overlap surface by an oil pollutant in the single-lap adhesive joints of aluminum plates.

The guided wave propagation method usually consists of the excitation of waves in one point of an analyzed structure and a collection of signals in some other points. If the number of measurement points (fitted with ultrasonic or piezoelectric transducers) is relatively small, the actual state of the considered structure is determined by the analysis of registered time histories. For a greater number of measurements, the non-contact methods are beneficial, allowing to sense the guided wave field in a considered area. The scanning laser Doppler vibrometry (SLDV) is one of the methods that provide a more accurate analysis [38,39,40,41]. As the effect, the plane representation of propagating waves (the so-called SLDV map) can be obtained. The existence of any defect in the scanned area results in the disturbance of the wave front shape, but its actual position and shape are indeterminable. Therefore, further signal processing is required to obtain a useful defect image. For example, Sohn et al. [42] detected delamination and disbond in composite plates based on the SLDV maps processed with the use of different techniques such as Laplacian image filtering. Another quite simple but effective method of damage imaging is based on the vibration energy distribution, and requires root mean square (RMS) calculations or its alternative weighted variant (WRMS). Recently, it has been successfully applied for the damage identification of different structures [43,44,45,46,47,48,49]. Saravanan et al. [43] detected missing bolts, attached masses, and openings in aluminum specimens assuming the excitation frequency as 50 kHz. Radzieński et al. [44] analyzed the detection of additional mass in aluminum and composite plates for frequencies of 35 and 10 kHz, respectively. In another work [45], they examined aluminum plates strengthened with riveted L-shape stiffeners, considering different excitation frequencies (5, 35, and 100 kHz). Aluminum plates with notches of different directions were studied by Lee and Park [46], who proved that the orientation of defects to the incident wave front was significant. In another research study, Lee et al. [47] investigated the notches and corrosion defects of different areas using the weighted root mean square and edge detection algorithms. Rucka et al. [48] studied the influence of a weighting factor on the efficiency of WRMS maps. Aryan et al. [49] visualized defects in the form of corrosion, surface cracks, and dents in aluminum plates and the delamination in a composite beam using scanning laser Doppler vibrometer and RMS calculations. The excitation frequencies were chosen from a range of 100 to 300 kHz. To sum up, the above-mentioned works present the application of root mean square calculations of registered guided wave signals without extensive consideration of the influence of the excitation frequency. This parameter was usually arbitrary assumed; notwithstanding, it can significantly affect the legibility of obtained RMS maps.

The aim of the study is damage imaging in a Lamb-wave based inspection of adhesively bonded joints. Particular attention was paid to the influence of the excitation frequency on the efficiency of obtained results. The guided wave signals were collected by the scanning laser Doppler vibrometer and further processed using root mean square calculations. The experimental research was conducted on a real-scale physical model of a single-lap joint of metal plates bonded with the epoxy-based adhesive. The verification of measurements was provided by numerical analyses carried out on finite element method (FEM) models. A novel element of the study is the proposition of choosing the adequate excitation frequency by the qualitative measure of the effectiveness of RMS damage imaging. The hypothesis is that an efficient frequency range for experimental measurements can be determined in the way of initial FEM calculations for artificial defects. The relative difference between RMS values in the damaged and intact areas of the joint can be assumed as the measure of the efficiency.

## 2. Materials and Methods

### 2.1. Specimen Description

**Figure 1:**
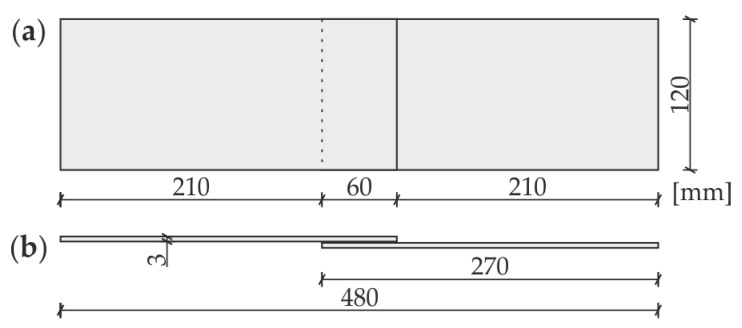
Geometry of investigated specimen: (**a**) plane view; (**b**) side view.

**Figure 2:**
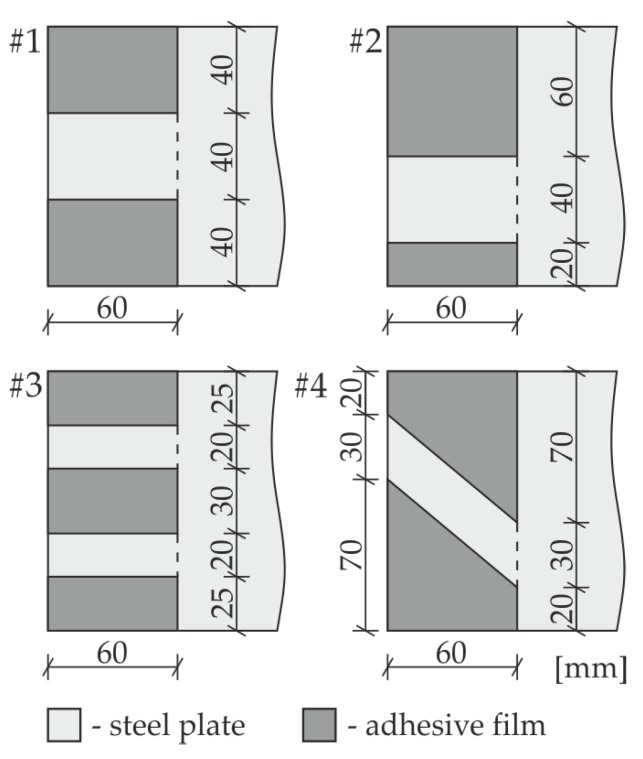
Variants of defects (#1 to #4).

**Figure 3:**
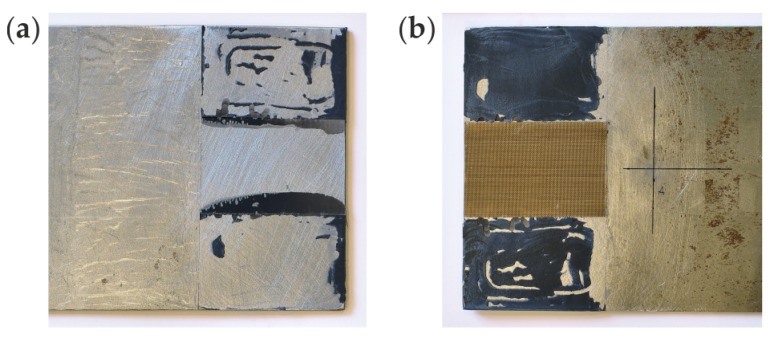
Photograph of experimental specimen after separation: (**a**) upper plate; (**b**) lower plate.

The investigations were conducted on the single-lap adhesive joints of steel plates. The geometry of the specimens is presented in Figure 1. The dimensions of each plate were 270 mm × 120 mm × 3 mm. The overlap surface was 120 mm × 60 mm. The internal defect in the form of partial debonding was designed in the adhesive film in four variants (#1 to #4, as shown in Figure 2), from which the first one was chosen for experimental measurements. The defect (#1) was obtained by sticking a PTFE (polytetrafluoroethylene) tape of 0.2-mm thickness in the middle of the overlap before manufacturing the connection. To avoid creating unintended debonding areas, the overlap surface of each adherend was treated with fine sandpaper (grit size 120) and degreased with Loctite-7063 cleaner just before joining. The epoxy-based adhesive Loctite Hysol 9461 (Henkel, Düsseldorf, Germany) was used to join the plates. The measured bondline thickness was equal to approximately 0.2 mm. To control the expected geometry of the defect, the adherends were disconnected after the experiments. The separated plates are presented in Figure 3. The failure occurred in the interfaces between the glue layer and the steel plates (mainly the lower one); it has a purely adhesive character. There are visible leaks of adhesive into the area of the intended defect (on the upper plate). Moreover, the defect edges are irregular (visible mainly on the lower plate).

### 2.2. Experimental Setup

**Figure 4:**
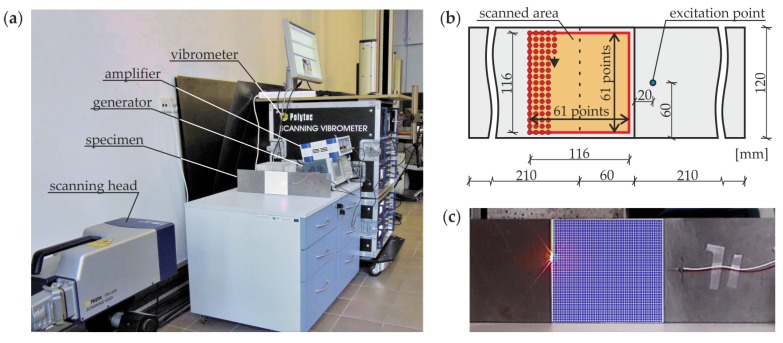
Experimental measurements: (**a**) experimental setup for the generation and acquisition of Lamb waves; (**b**) view of a specimen with the position of a scanned area and excitation point; (**c**) investigated specimen with indicated scanning points.

The experimental examination of prepared specimen #1 consisted of the excitation and the acquisition of the Lamb wave propagation signals in the specified area of the joint by the scanning laser Doppler vibrometry method. The experimental setup is presented in Figure 4a. The generation of the input wave signal was provided by the arbitrary function generator AFG 3022 (Tektronix, Inc., Beaverton, OR, USA) with the support of the high-voltage amplifier PPA 2000 (EC Electronics, Krakow, Poland). The plate piezoelectric actuator NAC2024 (Noliac, Kvistgaard, Denmark) with dimensions of 3 mm × 3 mm × 3 mm was used for the excitation of the guided wave field in one of the adherends. The actuator was attached to the top surface of the specimen by the petro wax 080A109 (PCB Piezotronics, Inc., Depew, NY, USA). The input signal was a wave packet obtained from the five periods of the sinusoidal function by the Hanning window modulation. The excitation frequency was individual for each measurement and varied from 20 to 350 kHz. The signals of the guided wave field were recorded by the scanning head of the laser vibrometer PSV-3D-400-M (Polytec GmbH, Berlin, Germany) equipped with a VD-07 velocity decoder. The sampling frequency was assumed to be 2.56 MHz. The improvement of light backscatter was provided by covering the scanned surface with a retro-reflective sheeting. The out-of-plane components of velocity values were acquired in the time domain in 3721 points distributed over the area featuring the overlap surface and the part of the plate after it (at the top side of the specimen); see Figure 4b,c. The scanning was performed point by point in the quadratic mesh of 61 rows and 61 columns, resulting in the resolution of about 1.93 mm. The representations of acquired signals for specific time instances show the propagation of the full guided wave field (SLDV maps).

### 2.3. FEM Modeling

**Figure 5:**
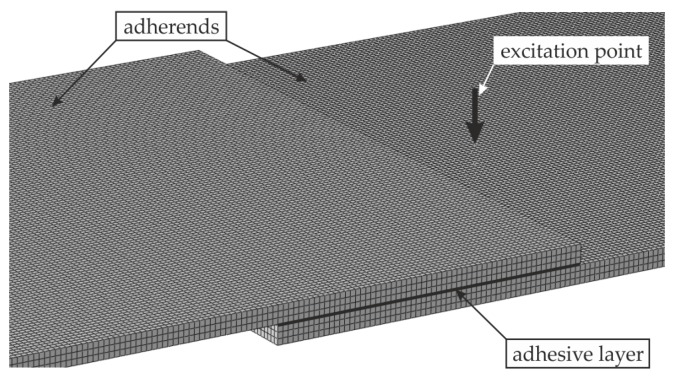
View of a discretized model for numerical calculations with an indicated excitation point.

The numerical modeling of elastic wave propagation in composite structures, such as adhesive joints, is a complex problem, mainly because of material inhomogeneity and an uncertainty of contact at the interfaces between different materials. An effective contribution to this issue was made by Chronopoulos [50] and Apalowo and Chronopoulos [51]. In the present paper, numerical analysis of the guided wave propagation in the considered adhesive joints (#1 to #4) was conducted using the finite element method in Abaqus/Explicit software. Some assumptions were made to simplify the modeling process and shorten the calculations. Three-dimensional FEM models were prepared for a transient dynamic analysis. Each structure was discretized by eight-node solid elements with reduced integration (C3D8R) from the explicit element library. The appropriate mapping of the wave behavior requires at least 20 nodes for the shortest wavelength of interest [52]. According to this limitation, the mesh was initially assumed to be regular and consisted of cube-shaped elements with a global size of 1 mm, which was reduced to 0.2 mm for the thickness of the adhesive layer (see Figure 5). The mesh convergence test was conducted taking into account a few refined meshes. The out-of-plane velocity values in some randomly chosen points at specific time instances were assumed as the measure of the convergence. The relative differences between results were negligible; thus, the exact calculations were conducted with the use of the above-mentioned mesh with the global element size of 1 mm. The boundary conditions were free at all the edges. The materials were adopted to meet the assumptions of a homogenous, isotropic material model. The material parameters were: for steel *E_s_* = 195.2 GPa, *ν_s_* = 0.30, *ρ_s_* = 7741.7 kg/m^3^, and for adhesive *E_a_* = 5 GPa, *ν_a_* = 0.35, and *ρ_a_* = 1330 kg/m^3^. The material damping was neglected because of its marginal influence on the RMS damage imaging. Both adherends and the adhesive film were assumed to be independent structures combined rigidly at the part of their surfaces by means of a tie connection (compatibility of translational degrees of freedom at all the contacting nodes). The excitation of guided waves was applied at the lower adherend in the form of the concentrated force surface load with the amplitude varying in time in accordance with the wave packet signal. The excitation frequency range was extended in comparison to experimental measurements (20 to 500 kHz). The dynamic analysis was conducted with the use of the central difference method with a fixed time step of 10^−7^ s. This value meets the recommendation of at least 20 points per each cycle of the wave with the higher frequency [52]. The results of the analysis were out-of-plane velocity signals collected at 3721 points spread over the area of the joint corresponding with experimental measurements.

### 2.4. RMS Damage Imaging

The signals of propagating waves acquired during scanning with a laser vibrometer need further processing techniques that allow detecting damaged areas and defining their actual shape. The essential point of damage imaging is to show the differences between the undamaged and damaged part of an analyzed structure. One of the simplest method consists of the calculation of the root mean square (RMS) for each recorded signal. The RMS value for the continuous time signal *s*(*t*) can be calculated with the following formula:

(1) $$RMS=\sqrt{\frac{1}{t_{2}-t_{1}}\int_{t_{1}}^{t_{2}}s{\left(t\right)}^{2}dt}$$

where *t*_1_ is the beginning and *t*_2_ is the end of the time window, which is defined as the difference between these two values. For a discrete signal *s_k_* = *s*(*t_k_*) recorded with the time interval ∆*t*, the RMS value can be calculated as follows:

(2) $$RMS=\sqrt{\frac{1}{n}\sum_{k=1}^{n}s_{k}^{2}}$$

where *n* is the number of samples, and the time window is defined as *T* = ∆*t*(*n* − 1) = *t*_2_ − *t*_1_. The map prepared from the calculated RMS values allows identifying and determining the geometry of any possible defects existing in the scanned area. Overall, for damaged areas of an analyzed element (e.g., delamination, crack, opening), different RMS values are attained because of different characteristics of Lamb wave propagation (changes due to material stiffness or geometry disturbance).

## 3. Results and Discussion

### 3.1. Dispersion Curves

**Figure 6:**
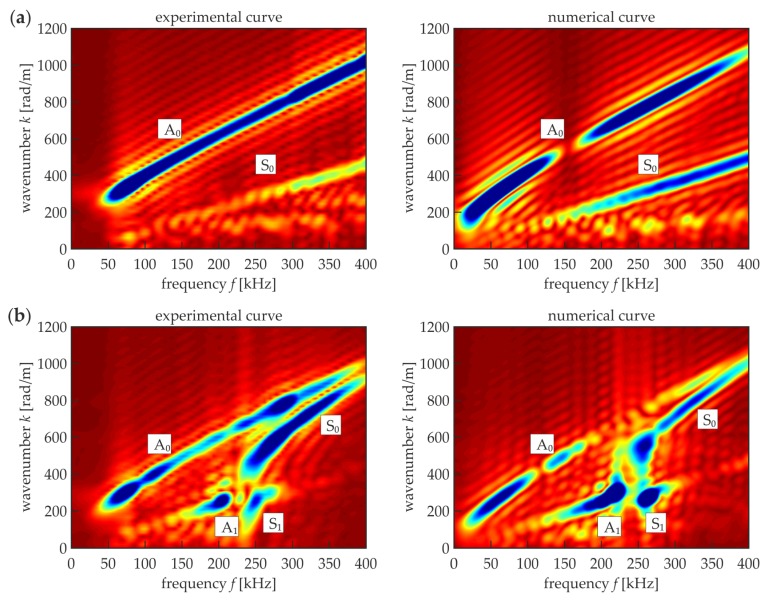
Experimental and numerical dispersion curves: (**a**) steel plate (sample D1)—single layer medium (*d_s_* = 3 mm, *E_s_* = 195.2 GPa, vs. = 0.3, *ρ_s_* = 7741.7 kg/m^3^); (**b**) adhesive joint (sample D2)—three-layer medium consisted of two steel plates (parameters same as in (**a**)) and adhesive film (*d_a_* = 0.2 mm, *E_a_* = 5 GPa, *v_a_* = 0.35, *ρ_a_* = 1330 kg/m^3^).

The initial step in the damage detection of adhesive joints was the comparison of Lamb wave characteristics in a three-layer medium (steel–adhesive–steel, simulating a properly prepared adhesive joint) and in a single-layer medium (single steel plate or disbonded area of the adhesive joint). For this purpose, dispersion curves were prepared experimentally and numerically for a steel plate with dimensions of 240 mm × 300 mm × 3 mm (sample D1) and for two plates with an adhesive film with a thickness of 0.2 mm bonding them together (sample D2). In each specimen, a wave packet in the form of a single-cycle Hanning windowed sinusoidal function was excited. The carrier frequency was changing in the range from 50 to 300 kHz with a step of 50 kHz. Additionally, for each frequency, symmetric and antisymmetric Lamb modes were excited independently. The velocity signals (out-of-plane components) were acquired in 101 points distributed along the straight line with a total length of 100 mm. The dispersion curves in the form of the maps representing wavenumber–frequency relations were obtained in the way of 2D-FFT (two-dimensional fast Fourier transform) calculations for each of 12 measurements (cf. [6,36,53]). The final result was the superposition of all the compound maps (Figure 6).

**Figure 7:**
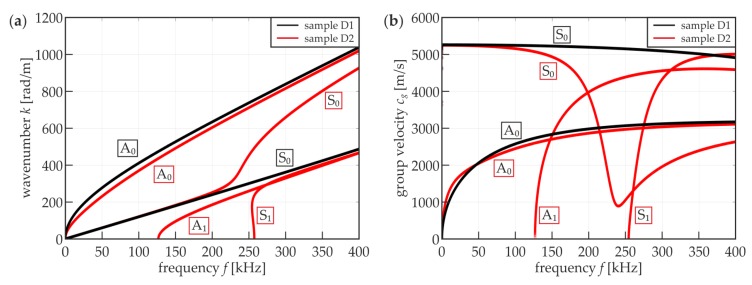
Theoretical dispersion curves for a steel plate (D1, black curves)–single layer medium (*d_s_* = 3 mm, *E_s_* = 195.2 GPa, vs. = 0.3, *ρ_s_* = 7741.7 kg/m^3^) and adhesive joint (D2, red curves)–three-layer medium consisting of two steel plates (parameters same as above) and adhesive film (*d_a_* = 0.2 mm, *E_a_* = 5 GPa, *v_a_* = 0.35, *ρ_a_* = 1330 kg/m^3^): (**a**) wavenumber–frequency relations; (**b**) group velocity–frequency relations.

A comparison of experimental and numerical curves led to the conclusion that both approaches gave consistent results. This also proved the appropriateness of the assumption of adhesive material parameters. To track the theoretical dispersion curves of Lamb waves in the investigated media, our own code was developed in the Matlab^®^ software (9.3.0.713597, The MathWorks, Inc., Natick, MA, USA), implementing the transfer matrix method [54,55]. Figure 7a shows wavenumber–frequency relations for both media (samples D1 and D2). The shape of the curves is approximately the same as that shown in the maps in Figure 6. However, the possibility of the effective excitation of certain modes was not the same in the results of the measurements (in both experimental and numerical curves for the single plate and the joint). In sample D1 (Figure 6a and Figure 7, black curves), only fundamental S_0_ and A_0_ modes can propagate in the considered frequency range, notwithstanding that the S_0_ curve is not as strongly exposed as A_0_ in the maps, which may be the result of the acquisition of only out-of-plane components on the upper surface that are related mainly to antisymmetric modes. In sample D2 (Figure 6b and Figure 7, red curves), in addition to the fundamental pair, A_1_ and S_1_ modes are present starting from the frequencies of about 130 and 260 kHz, respectively. Moreover, the shape of the S_0_ curve changes meaningfully compared with the single-layer plate. The shape of the A_0_ curve does not change significantly in comparison to sample D1. The differences between the two types of media are also clearly visible on group velocity–frequency relations (Figure 7b), which will be useful in further considerations.

### 3.2. Influence of Excitation Frequency on the RMS Damage Imaging

The analysis of the influence of the excitation frequency on the effectiveness of RMS damage imaging was performed for specimen #1. Experimental and numerical approaches were applied for an analysis of guided Lamb wave fields and RMS maps.

#### 3.2.1. Guided Wave Fields

**Figure 8:**
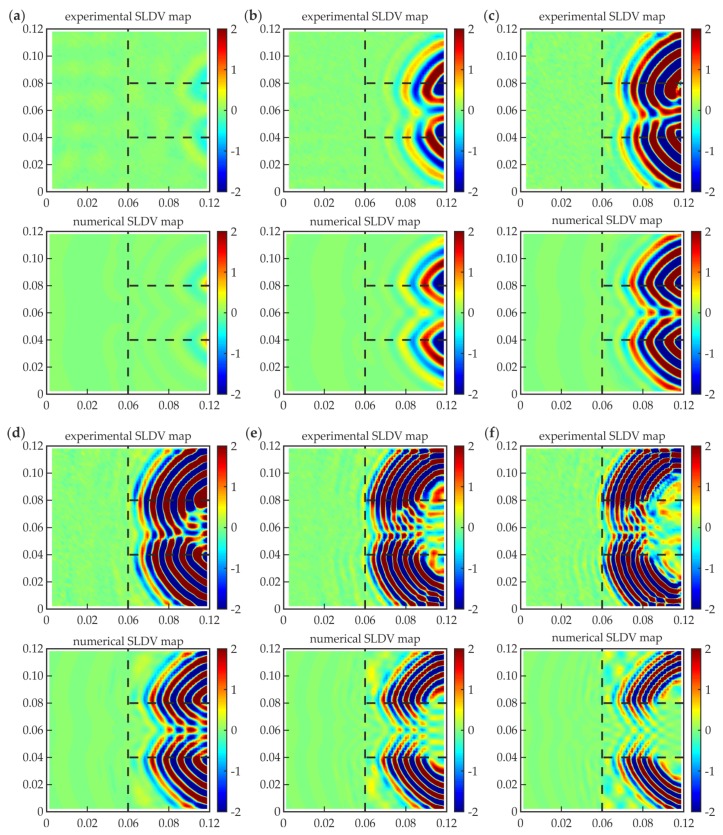
Experimental and numerical guided wave fields (values in m/s·10^−3^) for a specific time, 30 μs, and different excitation frequencies: (**a**) *f* = 50 kHz; (**b**) *f* = 100 kHz; (**c**) *f* = 150 kHz; (**d**) *f* = 210 kHz; (**e**) *f* = 300 kHz; and (**f**) *f* = 350 kHz.

Guided wave fields representing out-of-plane velocity values were prepared for a specific time instance *t* = 30 μs. Certain frequencies (50, 100, 150, 210, 300, and 350 kHz) were chosen for a comparative analysis of experimental and numerical results. The maps of propagating waves are presented in Figure 8. The comparison of presented snapshots revealed the variability of group velocity in relation to the excitation frequency. For the lowest frequency (50 kHz, Figure 8a), the wavefront is moderately visible (disturbance only at the initial part of the overlap). In the case of higher frequencies, the wavefront moved to the left side of the overlap, which suggests the greater speed of the excited wave packet. In fact, the individual selection of the time instance for each measurement can reduce these differences. Minor differences were observed between higher frequencies, because the group velocity was similar. Knowing that the excitation has an antisymmetric character, the A_0_ mode is expected to be dominant. These observations agree with the dispersion curves (Figure 7b). The A_0_ curve for the three-layer plate indicates significant growth in the group velocity value in the initial frequency range, and almost no variations in the further range. This explains why there are meaningful differences between snapshots for lower frequencies (50, 100, and 150 kHz), but wave fields are comparable for higher ones (210, 300, and 350 kHz), neglecting considerable changes in periods of wave packets.

Comparing the experimental and numerical results, there are some slight differences. Firstly, the numerical maps are symmetric, whereas the symmetry of the experimental wave fields is vaguely disturbed, probably by an imperfect preparation of the specimen and an inaccurate assumption of the scanning area for measurements. Moreover, the wavefronts are disturbed sharply at the edges of the defect in the numerical snapshots, but this effect is not that demonstrable in the experimental results because of the irregularities in the shape of defect edges (cf. Figure 3). Additionally, the group velocity is slightly higher for the experimental maps. The reason might lie in the differences between the mechanical properties of both materials (steel, adhesive) or the geometry of plates and the adhesive film (especially thickness). The observation of each snapshot allows identifying the defect. Significant disturbances of the wavefront indicate the intended lack of the adhesive in the middle of the overlap. Nonetheless, the determination of the actual geometry of the damaged area is not possible, and additional signal processing is required.

#### 3.2.2. RMS Imaging

**Figure 9:**
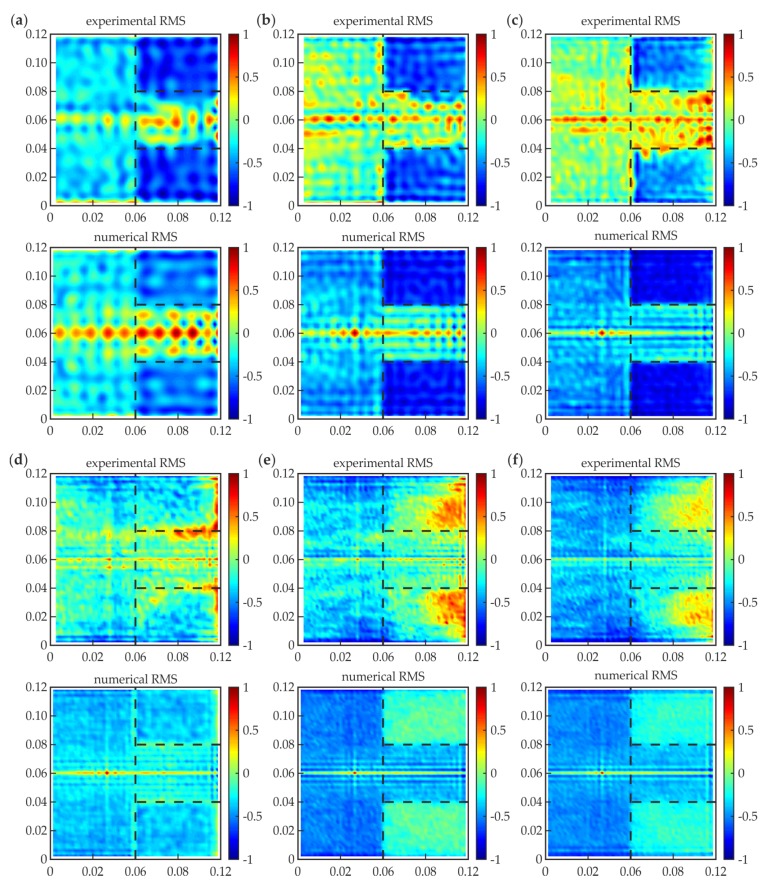
Experimental and numerical root mean square (RMS) maps for different excitation frequencies: (**a**) *f* = 50 kHz; (**b**) *f* = 100 kHz; (**c**) *f* = 150 kHz; (**d**) *f* = 210 kHz; (**e**) *f* = 300 kHz; and (**f**) *f* = 350 kHz.

Figure 9 shows the RMS maps normalized to unity for experimental and numerical signals collected for specimen #1. The chosen frequencies were the same as those for the SLDV maps. Each RMS value was calculated with respect to Equation (2). The time window covered the whole time of signal acquisition, i.e., *T* = 3.2 ms. The individual characterization of a single-layer medium (steel plate, such as the damage area) and a three-layer medium (properly prepared adhesive joint) should result in the clear difference of the calculated RMS values. However, it is undeniable that excitation frequency is an essential factor affecting the effectiveness of RMS damage imaging. For the lower frequencies (50 kHz, Figure 9a; 100 kHz, Figure 9b; 150 kHz, Figure 9c), the damaged area is characterized by highest RMS values rather than an appropriately prepared joint (similar to the single plate after the joint). The difference between these two areas is clear. Some differences between experimental and numerical maps result from irregularities in defect geometry (cf. Figure 3). It is worth noticing that at the lower excitation frequency, the lower resolution can be obtained in the map and, as a result, the larger defects can be omitted. This may be very important in the case of small defects; however, for the considered damage area, it is not essential. The frequency 210 kHz (Figure 9d) give an ineffectual result: there is almost no difference between the defect and intact joint, especially in the numerical map. In the experimental RMS, some boundary effects (intensification of the wave energy on the irregular edge) led to higher RMS values. This example shows that the invalid choice of the excitation frequency can make the measurement results useless. For the frequencies higher than 210 kHz (300 kHz, Figure 9e; 350 kHz, Figure 9f) the RMS values are lower in the damaged area than in the intact joint. The correlation between RMS values for these two areas is inverted. What is important is that the visual assessment of RMS maps shows that the distinction between the damaged and intact area of the joint is much more pronounced in the lower frequency range, especially for experimental maps, where the whole area of the adhesive layer does not have the same value. This may be the effect of the limitations of the used experimental setup. What is more, the increase in the excitation frequency is related to the increase in the wave attenuation.

The effectiveness of RMS damage identification is an important issue, so there is the need for a qualitative measure of contradistinction between the damaged and intact areas of the joint. The proposition is the relative difference between the level of the RMS in these two areas, which can be expressed by the relation:

(3) $$R_{d}=\frac{l_{d}-l_{i}}{l_{i}}$$

where *l_i_* and *l_d_* denote the mean RMS value in the properly prepared area of the overlap and in the damaged area, respectively. The definition of the *R_d_* value induces that the damaged area is defined, so it cannot be used if the joint has any unknown defects. Nevertheless, the aim of *R_d_* calculations is only the demonstration of changes in the effectiveness of RMS imaging in relation to the excitation frequency. The area of the overlap was divided before the calculations into two parts (damaged and intact) with a rejection of points localized on the edges of the defect and on the longitudinal axis of the joint, because for these points, the RMS values are distinctly high (intensification of energy evoked by the symmetry and boundaries, cf. Figure 9). The mean RMS values were calculated for the points of both areas, and the *R_d_* value was calculated for measurements over the whole considered range of frequency.

**Figure 10:**
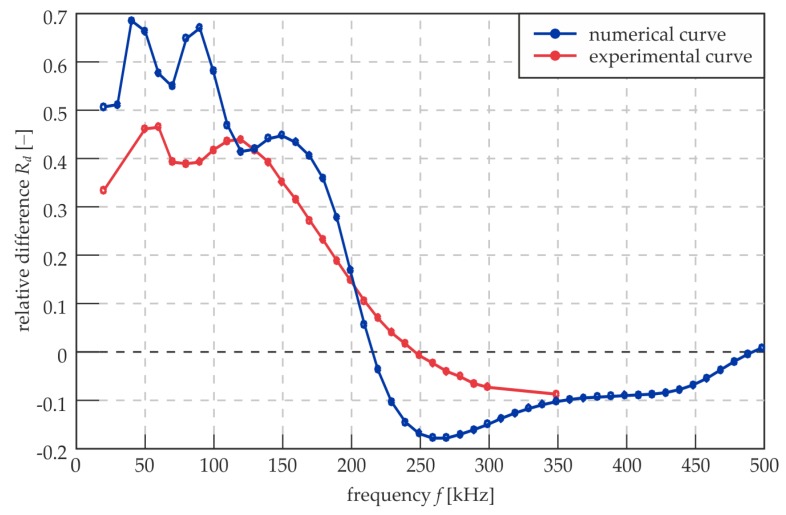
Relative difference between mean RMS values for damaged and intact areas of overlap for experimental (frequency range from 20 to 350 kHz) and numerical results (frequency range from 20 to 500 kHz).

Figure 10 shows the relation between *R_d_* and excitation frequency for experimental and numerical results. The curves are slightly different, but both have some characteristic points. The first one is a local maximum for 50 kHz. For this value, the A_0_ modes for single and three-layer plates are crossing on dispersion curves (cf. Figure 7b). Then, there are some fluctuations that differ between the two curves. Another peak repeating for both experimental and numerical curves is for about 120 kHz, when the A_1_ mode for the three-layer plate appears. Further, the curves are falling monotonically. The numerical curve has the root equal to approximately 215 kHz, and this is the frequency value for which the damaged area and intact joint are not distinguishable (cf. Figure 9). For the experimental curve, the root is translated to approximately 250 kHz. This may be the result of the energy intensification on the edges of the defect (cf. Figure 9d). The global minimum for the numerical curve is attained for approximately 260 kHz (the appearance of the S_1_ mode for the three-layer plate). This is the frequency for which the joint and the defect can be distinguished with maximal efficiency in the frequency range above 210 kHz. Further, the curve is slightly rising until obtaining another root (about 500 kHz) at the end of the frequency range. The experimental curve does not obtain the local minimum above 210 kHz; instead, it is constantly falling to the end of the assumed frequency range up to 350 kHz. Generally, the positive values of *R_d_* are obtained when the damaged area is characterized by higher RMS values than the intact joint. Negative values indicate on the inverted relation. If *R_d_* equals zero, there is no possibility of identifying the defects. What is important is that the absolute values of *R_d_* are higher below the first root (about 215 kHz), which suggests that the lower frequencies allow obtaining a better differentiation of three-layer and single layer media. However, the above-mentioned decrease in map resolution cannot be neglected. To compromise both of these factors (the differentiation between defect and intact joint and the map resolution), the excitation frequency should be chosen from an approximate range of 120 to 180 kHz. The determination of an optimal frequency value requires mathematical optimization and a proposition of an objective function containing components linked with the image resolution and the *R_d_* value.

#### 3.2.3. Statistical Analysis of RMS Values

In addition to the foregoing considerations, the statistical analysis was conducted. All the RMS values calculated for the whole overlap surface, rejecting points at the edges of the defect and on the longitudinal axis (as for *R_d_* calculations), were treated as the single series of values. Histograms were calculated for each dataset. If there are no defects in the analyzed area, the purely unimodal distribution would be obtained, because all the RMS values should accumulate over a single value, which is symbolized above by *l_i_*. The presence of a defect in the adhesive layer should result in the bimodal distribution caused by the existence of two dominant values for the intact joint *l_i_* and for defect *l_d_*.

**Figure 11:**
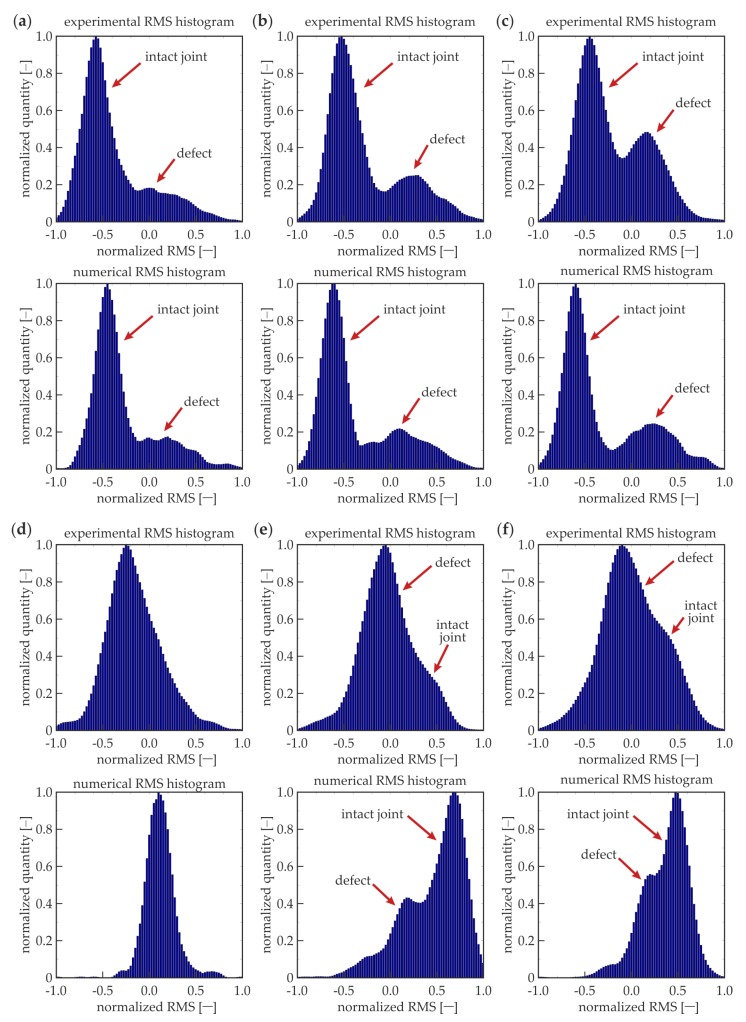
Experimental and numerical RMS histograms for different excitation frequencies: (**a**) *f* = 50 kHz; (**b**) *f* = 100 kHz; (**c**) *f* = 150 kHz; (**d**) *f* = 210 kHz; (**e**) *f* = 300 kHz; and (**f**) *f* = 350 kHz.

Figure 11 presents RMS histograms that have been prepared for certain frequencies (the same as for the RMS maps). The results are normalized, both for the RMS value and the quantity axes. For the lower frequencies (50, 100, and 150 kHz), bimodal distributions were obtained for the experimental and numerical data. The first mode is characterized by the lower RMS values and related to the intact joint area (cf. Figure 9a–c). The second mode indicates the defect existence (higher RMS values), and it obtains less quantity than the first mode, because the defect surface is twice as small as the intact joint surface. For 210 kHz (Figure 9d), the histograms are unimodal, which results from the equality of mean RMS values calculated for the defect and intact joint (*R_d_* = 0, not efficient damage imaging). The experimental histogram is not as narrow as the numerical one, which is related to the translation of root of curves from Figure 10. For higher frequencies (300 kHz, 350 kHz), the distributions are not unimodal; the damage can be identified, and its mode is related to the lower RMS values. The dissociation of modes is not as clear as for lower frequencies—this effect is related to the lower absolute values of *R_d_* for higher frequencies. In numerical histograms, the defect mode is characterized by the lower intensity than the intact joint mode (compatibly with the relation of surfaces of damaged and intact areas). The experimental histograms do not cover the same rule; the intact joint mode has a lower intensity because higher RMS values are not obtained in the whole area of the properly prepared joint (cf. Figure 9e,f). To sum up, the histogram analysis can reveal the existence of damaged areas, but it has some limitations. Firstly, the geometry of the defects cannot be determined. Moreover, the method is not efficient for small defects, because the damage modes would be of small quantity, which makes it impossible to identify them on the histograms.

### 3.3. Influence of Different Defect Geometry

**Figure 12:**
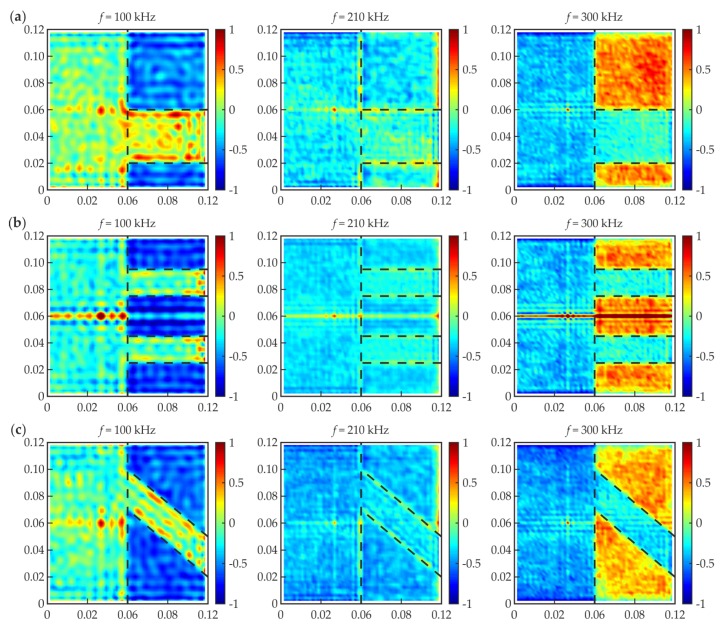
Numerical RMS maps for different excitation frequencies (100, 210, and 300 kHz) and different defects: (**a**) #2; (**b**) #3; and (**c**) #4.

**Figure 13:**
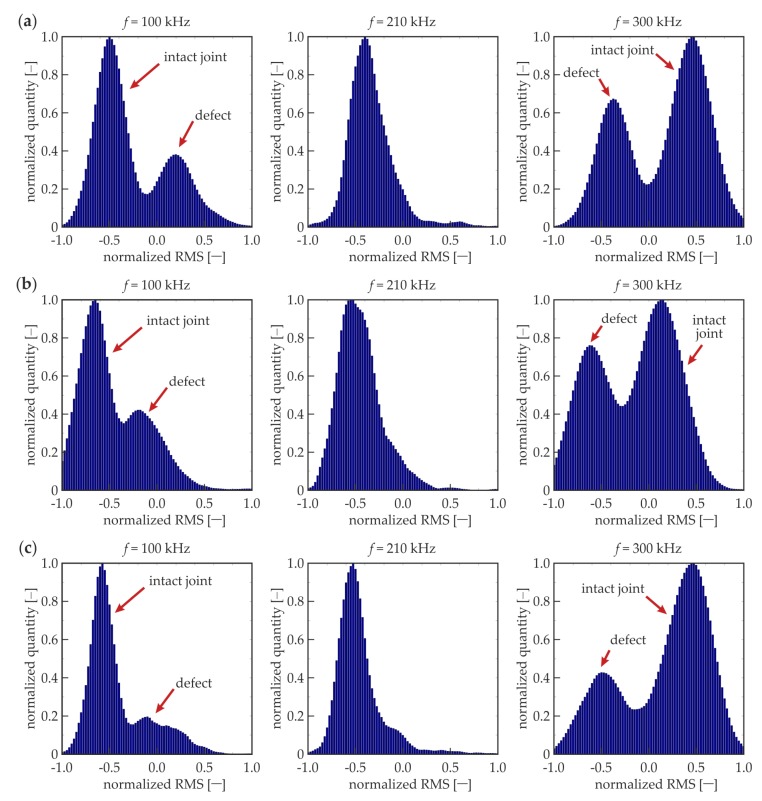
Numerical RMS histograms for different excitation frequencies (100, 210, and 300 kHz) and different defects: (**a**) #2; (**b**) #3; and (**c**) #4.

The above considerations were conducted only for a single joint, #1. Next, the observed effects were verified on specimens #2 to #4 by the way of numerical calculations for three certain frequencies (100 kHz, 210 kHz, and 300 kHz). The normalized RMS maps are presented in Figure 12. It is visible that for the frequency of 100 kHz, higher RMS values were obtained for the damaged areas than for the intact joint (for all the analyzed specimens). The frequency of 210 kHz appeared to be inefficient for RMS damage imaging, i.e., the difference between the damaged and properly prepared area was not significant. The frequency of 300 kHz resulted in lower RMS values in the damaged area. Moving to the histograms (Figure 13), the frequency 210 kHz gave the unimodal distribution (no difference between the defect and intact joint). The histograms for 100 and 300 kHz gave bimodal distributions, but for lower frequencies, the defect mode was related to higher RMS values, whereas for higher frequencies, it was related to lower RMS values. The quantity for the defect mode was always smaller than that for an intact joint. The considerations for joints #2 to #4 provided the same results as for specimen #1. Summarizing, the efficiency of a measurement with a specific excitation frequency does not change with the geometry of a damaged area. It is a satisfying conclusion, because generally, the geometry of the damaged area is unknown. This means that an additional preliminary study consisting of numerical calculations can reveal the appropriate excitation frequency value and reduce the number of measurements. However, the possibility of the damage identification due to a frequency value depends strongly on the characteristics of the considered media, so they need to be determined.

## 4. Conclusions

The paper discussed the effects of the wave frequency on the efficiency of damage detection in adhesive joints of steel plates using Lamb wave propagation and RMS imaging. Experimental and numerical approaches were applied. The research comprised the visual appreciation of obtained RMS maps and statistical analysis of calculated values. The study resulted in the conclusions presented below.

- The guided wave fields enabled identifying the occurrence of the defect regardless of the excitation frequency. However, the actual location and shape are indeterminable; thus, guided wave field measurements can only be an initial step for further analyses.
- The RMS maps allowed determining the geometry of the damaged areas. The effectiveness of damage visualization was strongly dependent on the excitation frequency.
- The variability of the relative difference between the mean RMS values for the intact joint and the damage was fully compatible with the clarity of the RMS maps. Some analogies between the relative RMS difference and dispersion curves were observed.
- The statistical analysis was successfully used to determine the effectiveness of the results obtained for different excitation frequencies based on the RMS histograms. The important advantage of this approach is the independence of the defect geometry.
- The statistical analysis in a certain frequency range on the single numerical model with a random defect can be sufficient for the determination of the adequate frequency for the further experimental testing of samples with an unknown state.

The guiding conclusion was that the Lamb wave-based inspection of adhesive joints with the use of scanning laser Doppler vibrometry and signal processing, such as root mean square calculations, provides a successful method for damage imaging. To obtain valuable results, some initial analyses need to be conducted before the exact measurements. The main factor is the choice of an appropriate excitation frequency, which can be conducted using numerical calculations supported by statistical analysis.

## References

[1] Adams R.D., Wake W.C. (1986). Structural Adhesive Joints in Engineering.

[2] Dillard D.A. (2010). Advances in Structural Adhesive Bonding.

[3] Martínez-Landeros V.H., Vargas-Islas S.Y., Cruz-González C.E., Barrera S., Mourtazov K., Ramírez-Bon R. (2019). Studies on the influence of surface treatment type, in the effectiveness of structural adhesive bonding, for carbon fiber reinforced composites. J. Manuf. Process..

[4] Jeenjitkaew C., Guild F.J. (2017). The analysis of kissing bonds in adhesive joints. Int. J. Adhes. Adhes..

[5] Sengab A., Talreja R. (2016). A numerical study of failure of an adhesive joint influenced by a void in the adhesive. Compos. Struct..

[6] Ong W.H., Rajic N., Chiu W.K., Rosalie C. (2018). Lamb wave–based detection of a controlled disbond in a lap joint. Struct. Health Monit..

[7] Ren B., Lissenden C.J. (2013). Ultrasonic guided wave inspection of adhesive bonds between composite laminates. Int. J. Adhes. Adhes..

[8] Puthillath P.K., Yan F., Kannajosyula H. Inspection of Adhesively Bonded Joints Using Ultrasonic Guided Waves. Proceedings of the 17th World Conference on Nondestructive Testing.

[9] Korzeniowski M., Piwowarczyk T., Maev R.G. (2014). Application of ultrasonic method for quality evaluation of adhesive layers. Arch. Civ. Mech. Eng..

[10] Tighe R.C., Dulieu-Barton J.M., Quinn S. (2016). Identification of kissing defects in adhesive bonds using infrared thermography. Int. J. Adhes. Adhes..

[11] Opdam N.J.M., Roeters F.J.M., Verdonschot E.H. (1997). Adaptation and radiographic evaluation of four adhesive systems. J. Dent..

[12] Sato T., Tashiro K., Kawaguchi Y., Ohmura H., Akiyama H. (2019). Pre-bond surface inspection using laser-induced breakdown spectroscopy for the adhesive bonding of multiple materials. Int. J. Adhes. Adhes..

[13] Steinbild P.J., Höhne R., Füßel R., Modler N. (2019). A sensor detecting kissing bonds in adhesively bonded joints using electric time domain reflectometry. NDT E Int..

[14] Malik H., Zatar W. (2018). Software Agents to Support Structural Health Monitoring (SHM)-Informed Intelligent Transportation System (ITS) for Bridge Condition Assessment. Procedia Comput. Sci..

[15] dos Reis J., Oliveira Costa C., Sá da Costa J. (2019). Local validation of structural health monitoring strain measurements. Meas. J. Int. Meas. Confed..

[16] Comisu C.C., Taranu N., Boaca G., Scutaru M.C. (2017). Structural health monitoring system of bridges. Procedia Eng..

[17] Yang J.P., Chen W.Z., Li M., Tan X.J., Yu J.X. (2018). Structural health monitoring and analysis of an underwater TBM tunnel. Tunn. Undergr. Space Technol..

[18] Miśkiewicz M., Pyrzowski Ł., Wilde K., Mitrosz O. (2017). Technical Monitoring System for a New Part of Gdańsk Deepwater Container Terminal. Polish Marit. Res..

[19] Gomes G.F., Mendéz Y.A.D., da Silva Lopes Alexandrino P., da Cunha S.S., Ancelotti A.C. (2018). The use of intelligent computational tools for damage detection and identification with an emphasis on composites—A review. Compos. Struct..

[20] Martins A.T., Aboura Z., Harizi W., Laksimi A., Khellil K. (2019). Structural health monitoring for GFRP composite by the piezoresistive response in the tufted reinforcements. Compos. Struct..

[21] Chroscielewski J., Miskiewicz M., Pyrzowski L., Rucka M., Sobczyk B., Wilde K., Pakzad S. (2019). Dynamic Tests and Technical Monitoring of a Novel Sandwich Footbridge. Dynamics of Civil Structures, Volume 2.

[22] Ostachowicz W., Kudela P., Krawczuk M., Zak A. (2012). Guided Waves in Structures for SHM: The Time-Domain Spectral Element Method.

[23] Rose J.L. (2014). Ultrasonic Guided Waves in Solid Media.

[24] Yu X., Zuo P., Xiao J., Fan Z. (2019). Detection of damage in welded joints using high order feature guided ultrasonic waves. Mech. Syst. Signal Process..

[25] Zhang W., Hao H., Wu J., Li J., Ma H., Li C. (2018). Detection of minor damage in structures with guided wave signals and nonlinear oscillator. Meas. J. Int. Meas. Confed..

[26] Pan W., Sun X., Wu L., Yang K., Tang N. (2019). Damage Detection of Asphalt Concrete Using Piezo-Ultrasonic Wave Technology. Materials (Basel).

[27] Schabowicz K. (2014). Ultrasonic tomography - The latest nondestructive technique for testing concrete members - Description, test methodology, application example. Arch. Civ. Mech. Eng..

[28] He S., Ng C.T. (2017). Guided wave-based identification of multiple cracks in beams using a Bayesian approach. Mech. Syst. Signal Process..

[29] Pahlavan L., Blacquière G. (2016). Fatigue crack sizing in steel bridge decks using ultrasonic guided waves. NDT E Int..

[30] Munian R.K., Mahapatra D.R., Gopalakrishnan S. (2018). Lamb wave interaction with composite delamination. Compos. Struct..

[31] Shoja S., Berbyuk V., Boström A. (2018). Delamination detection in composite laminates using low frequency guided waves: Numerical simulations. Compos. Struct..

[32] Xiao H., Shen Y., Xiao L., Qu W., Lu Y. (2018). Damage detection in composite structures with high-damping materials using time reversal method. Nondestruct. Test. Eval..

[33] Nicassio F., Carrino S., Scarselli G. (2019). Elastic waves interference for the analysis of disbonds in single lap joints. Mech. Syst. Signal Process..

[34] Sunarsa T.Y., Aryan P., Jeon I., Park B., Liu P., Sohn H. (2017). A reference-free and non-contact method for detecting and imaging damage in adhesive-bonded structures using air-coupled ultrasonic transducers. Materials (Basel).

[35] Parodi M., Fiaschi C., Memmolo V., Ricci F., Maio L. (2019). Interaction of Guided Waves with Delamination in a Bilayered Aluminum-Composite Pressure Vessel. J. Mater. Eng. Perform..

[36] Gauthier C., Ech-Cherif El-Kettani M., Galy J., Predoi M., Leduc D., Izbicki J.L. (2017). Lamb waves characterization of adhesion levels in aluminum/epoxy bi-layers with different cohesive and adhesive properties. Int. J. Adhes. Adhes..

[37] Castaings M. (2014). SH ultrasonic guided waves for the evaluation of interfacial adhesion. Ultrasonics.

[38] Kudela P., Wandowski T., Malinowski P., Ostachowicz W. (2016). Application of scanning laser Doppler vibrometry for delamination detection in composite structures. Opt. Lasers Eng..

[39] Rothberg S.J., Allen M.S., Castellini P., Di Maio D., Dirckx J.J.J., Ewins D.J., Halkon B.J., Muyshondt P., Paone N., Ryan T. (2017). An international review of laser Doppler vibrometry: Making light work of vibration measurement. Opt. Lasers Eng..

[40] Derusova D., Vavilov V., Sfarra S., Sarasini F., Krasnoveikin V., Chulkov A., Pawar S. (2019). Ultrasonic spectroscopic analysis of impact damage in composites by using laser vibrometry. Compos. Struct..

[41] Pieczonka Ł., Ambroziński Ł., Staszewski W.J., Barnoncel D., Pérès P. (2017). Damage detection in composite panels based on mode-converted Lamb waves sensed using 3D laser scanning vibrometer. Opt. Lasers Eng..

[42] Sohn H., Dutta D., Yang J.Y., Desimio M., Olson S., Swenson E. (2011). Automated detection of delamination and disbond from wavefield images obtained using a scanning laser vibrometer. Smart Mater. Struct..

[43] Saravanan T.J., Gopalakrishnan N., Rao N.P. (2015). Damage detection in structural element through propagating waves using radially weighted and factored RMS. Measurement.

[44] Radzieński M., Doliński L., Krawczuk M., Zak A., Ostachowicz W. (2011). Application of RMS for damage detection by guided elastic waves. J. Phys. Conf. Ser..

[45] Radzieński M., Doliński Ł., Krawczuk M., Palacz M. (2013). Damage localisation in a stiffened plate structure using a propagating wave. Mech. Syst. Signal Process..

[46] Lee C., Park S. (2014). Flaw Imaging Technique for Plate-Like Structures Using Scanning Laser Source Actuation. Shock Vib..

[47] Lee C., Zhang A., Yu B., Park S. (2017). Comparison study between RMS and edge detection image processing algorithms for a pulsed laser UWPI (Ultrasonic wave propagation imaging)-based NDT technique. Sensors (Switzerland).

[48] Rucka M., Wojtczak E., Lachowicz J. (2018). Damage imaging in Lamb wave-based inspection of adhesive joints. Appl. Sci..

[49] Aryan P., Kotousov A., Ng C.T., Cazzolato B.S. (2017). A baseline-free and non-contact method for detection and imaging of structural damage using 3D laser vibrometry. Struct. Control Health Monit..

[50] Chronopoulos D. (2018). Calculation of guided wave interaction with nonlinearities and generation of harmonics in composite structures through a wave finite element method. Compos. Struct..

[51] Apalowo R.K., Chronopoulos D. (2019). A wave-based numerical scheme for damage detection and identification in two-dimensional composite structures. Compos. Struct..

[52] Moser F., Jacobs L.J., Qu J. (1999). Modeling elastic wave propagation in waveguides with the finite element method. NDT E Int..

[53] Gauthier C., Galy J., Ech-Cherif El-Kettani M., Leduc D., Izbicki J.L. (2018). Evaluation of epoxy crosslinking using ultrasonic Lamb waves. Int. J. Adhes. Adhes..

[54] Lowe M.J.S. (1995). Matrix Techniques for Modeling Ultrasonic-Waves in Multilayered Media. IEEE Trans. Ultrason. Ferroelectr. Freq. Control.

[55] Maghsoodi A., Ohadi A., Sadighi M. (2014). Calculation of Wave Dispersion Curves in Multilayered Composite-Metal Plates. Shock Vib..

